# Premature T cell aging in major depression: A double hit by the state of disease and cytomegalovirus infection

**DOI:** 10.1016/j.bbih.2023.100608

**Published:** 2023-02-27

**Authors:** Maria S. Simon, Magdalini Ioannou, Gara Arteaga-Henríquez, Annemarie Wijkhuijs, Raf Berghmans, Richard Musil, Norbert Müller, Hemmo A. Drexhage

**Affiliations:** a Department of Psychiatry and Psychotherapy, University Hospital, Ludwig-Maximilians-University, 80336, Munich, Germany; b Department of Psychiatry, University Medical Center Groningen, University of Groningen, Groningen, 9713, GZ, Netherlands; c Department of Psychiatry, Hospital Universitari Vall d'Hebron, Vall d'Hebron Research Institute (VHIR), Vall d'Hebron Barcelona Hospital Campus, Barcelona, Spain; d Biomedical Network Research Centre on Mental Health (CIBERSAM), Madrid, Spain; e Department of Immunology, Erasmus Medical Center, Rotterdam, 3015, GD, Netherlands; f Advanced Practical Diagnostics BVBA, Turnhout, 2300, Belgium

**Keywords:** Major depressive disorder, T helper cell, T cytotoxic cell, Senescence, CMV, Childhood trauma

## Abstract

**Introduction:**

Previous research indicates that premature T cell senescence is a characteristic of major depressive disorder (MDD). However, known senescence inducing factors like cytomegalovirus (CMV) infection or, probably, childhood adversity (CA) have not been taken into consideration so far.

**Objective:**

Differentiation and senescent characteristics of T cells of MDD patients were investigated in relation to healthy controls (HC), taking the CMV seropositivity and CA into account.

**Methods:**

127 MDD and 113 HC of the EU-MOODSTRATIFICATION cohort were analyzed. Fluorescence activated cell sorting (FACS) analysis was performed to determine B, NK, and T cell frequencies. In a second FACS analysis, naïve, effector memory (Tem), central memory (Tcm), effector memory cells re-expressing RA (TEMRA), as well as CD28^+^ and CD27^+^ memory populations, were determined of the CD4^+^ and CD8^+^ T cell populations in a subsample (N = 35 MDD and N = 36 HC). CMV-antibody state was measured by IgG ELISA and CA by the Childhood Trauma Questionnaire.

**Results:**

We detected a CMV-antibody positivity in 40% of MDD patients (35% HC, n. s.) with seropositive MDD cases showing a higher total childhood trauma score. Second, a higher inflation of memory CD4^+^ T helper cells in CMV seronegative patients as compared to seronegative HC and reduced numbers of naïve CD4^+^ T helper cells in CMV seropositive patients (not in CMV seropositive HC) were found. Third, a higher inflation of memory CD8^+^ T cytotoxic cells in CMV seropositive cases as compared to CMV seropositive HC, particularly of the TEMRA cells, became apparent. Higher percentages of CD4^+^ TEMRA and late stage CD27^−^CD28^−^ TEMRA cells were similar in both HC and MDD with CMV seropositivity. Overall, apportioning of T cell subpopulations did not differ between CA positive vs negative cases.

**Conclusions:**

MDD patients show several signs of a CMV independent “MDD specific” premature T cell aging, such as a CMV independent increase in CD4^+^ T memory cells and a latent naïve CD4 T-cell reduction and a latent CD8^+^ T-cell increase. However, these two latent T cell senescence abnormalities only become evident with CMV infection (double hit).

## Highlights

• Increased T helper memory cells irrespective of CMV seropositivity in MDD.
• Latent decrease in T helper naïve cells uncovered by CMV seropositivity in MDD.
• Latent increase in T cytotoxic memory cells uncovered by CMV seropositivity in MDD.
• Childhood trauma did not have effects on T cell senescence in MDD.

## 1 Introduction

Major Depressive Disorder (MDD) is a prevalent disorder with a heterogeneous array of symptoms. Evidence is accumulating that immune dysfunction plays an important role in this heterogeneity, and perhaps even in the causation of MDD (Dantzer et al., 2008; Irwin and Miller, 2007; Miller, 2010; Raison and Miller, 2011). Many studies have been published on abnormalities on the level of pro-inflammatory cytokines in the circulation of patients (Köhler et al., 2017) and have been interpreted as a sign of low-grade inflammation, affecting gray and white matter function (Gibney and Drexhage, 2013; Branchi et al., 2021). There is, however, a relative paucity of studies on abnormalities in the apportioning and function of monocytes and subsets of lymphocytes in the circulation of patients, while these cells are also important for brain function (Beumer et al., 2012; Poletti et al., 2017).

We recently published a study showing the expression of inflammation related genes in circulating monocytes of MDD patients of the EU-MOODSTRATIFICATION cohort (www.moodstrtaification.eu) (Simon et al., 2021). Outcomes of this monocyte study pointed toward a premature aging (senescence) of the cells in MDD, while in patients with a history of childhood adversity (CA) the senescent monocytes showed an additionally increased inflammatory state with higher expression of genes for typical pro-inflammatory cytokines such as IL-1 and IL-6. In studies on the apportioning of lymphocyte subsets in the circulation of patients of the EU-MOODSTRATIFICATION cohort we also found similar signs of premature aging, but then related to the CD4^+^ T helper compartment (Schiweck et al., 2020). Increased CD45RO + T helper memory cells were found in the MDD patients as compared to healthy controls (HC), also during young adulthood (Schiweck et al., 2020). In that study we concluded that future lymphocyte studies on our MDD cohort should take more detailed parameters for premature immune aging into account, such as anti-cytomegalovirus (CMV) titers (as a sign of chronic CMV infection) and percentages of end-stage differentiated senescent CD4+T helper and CD8+T cytotoxic cells. Chronic CMV infection is associated with accelerated immuno-senescence, while having outspoken effects on senescence characteristics of the CD8^+^ cytotoxic T cell compartment, but with lesser effects on the CD4^+^ T helper cell population (Nikolich-Žugich and van Lier, 2017; Pawelec et al., 2009; Weltevrede et al., 2016). Moreover, chronic CMV infection has been associated with depression (Gale et al., 2018). With regard to differentiation characteristics relevant for T cell aging, memory T cells appear in different forms (Abbas et al., 2021). We recognize central memory T cells (recirculating in the lymphoid compartment and playing a role in long duration memory), effector memory T cells (capable of migrating to the tissues and involved in immediate combat with the microbes) and effector memory T cells re-expressing RA (TEMRA; a further differentiated form of effector memory cells). Particularly in the TEMRA population T cells may show signs of exhaustion, which is a poor responsiveness to antigen presenting cells and characterized by an absence of CD28 and positivity for CD57. This population is often referred to as end-stage differentiated memory T cells. While the total repertoire of memory T cells provides a broad spectrum of antigen specificities, exhausted end-stage TEMRA cells are often monoclonal expansions specific for only a few immunodominant antigens which are predominantly derived from persistent viral infections, such as CMV (Derhovanessian et al., 2011; Tian et al., 2017). These cells have a poor proliferative capacity, a high sensitivity to apoptosis, are pro-inflammatory, and - in the case of CD8^+^ T cells - highly cytotoxic (Geginat et al., 2003; Hamann et al., 1997; Sallusto et al., 1999).

In the present study the differentiation and senescent characteristics of the T cells of the MDD patients of the EU-MOODSTRATIFICATION cohort are investigated in more detail, also taking into account the seropositivity of MDD patients and HC for CMV as a sign of chronic infection. For this purpose, we first re-analyzed the previous T cell staining (staining A and staining B of Schiweck et al., 2020) taking the CMV-antibody state of the patients and controls into account. Thereafter, we performed an additional FACS analysis (staining C) in the CD4^+^ and CD8^+^ populations of the patients and HC for the determination of naïve (CCR7+CD45RA+), effector memory (Tem, CCR7^neg^CD45RA^neg^), central memory (Tcm, CCR7+CD45RA^neg^), and effector memory cells re-expressing RA (TEMRA, CCR7^neg^CD45RA+). In the memory populations we also investigated the expression of CD28 and CD27. We studied whether CMV-antibody state and CA had an impact on the apportioning of the T lymphocyte subpopulations and senescence characteristics of T cells.

## 2 Methods

### 2.1 Participants

Using a cross-sectional design, participants were recruited at the three psychiatric university hospital sites Munich, Münster, and Leuven. The MDD group comprised N = 127 and the HC group N = 113 participants for whom CMV titer determinations were available. The present investigation is a continuation of the work by Schiweck et al. (2020) using the same staining data (staining A and B). For part of the investigation, a subset of the population was drawn according to the availability of lab samples to carry out an addition staining C. Female and male adults aged 18–65 years were included. MDD patients had to be free of the following diseases: clinical inflammation-related symptoms (including fever), current or recent inflammatory or infectious disease, uncontrolled systemic disease, uncontrolled metabolic disease, other uncontrolled somatic disorder affecting mood. Additionally, patients were excluded if they used somatic medication affecting mood or the immune system, e.g., statins, corticosteroids, non-steroidal anti-inflammatory drugs.

HC were excluded if they were not in self-declared health (specifically lacking any form of auto-immune disease and/or atopic disease) and/or used somatic medication that affects mood or the immune system. In both groups, pregnant women or women who had delivered within the previous 6 months were excluded.

Presence of MDD was diagnosed by the Mini-International Neuropsychiatric Interview (MINI; Sheehan et al., 1998). Healthy controls were screened for absence of psychiatric symptoms using the MINI Screening version. The study was conducted in accordance with the declaration of Helsinki and its subsequent revisions and approved by the respective ethical committees of the participating universities (reference numbers: Leuven: S51723; Munich: 291–09, Münster: 2009-019-f-S). Written informed consent was obtained from all participants.

### 2.2 Clinical assessment

Childhood adverse events were measured by the Childhood Trauma Questionnaire (CTQ; Bernstein et al., 2003). The presence of trauma on at least one subscale of the CTQ, according to the criteria by Walker et al. (1999), defined childhood adversity (CA) positive cases. The cut-offs for each scale were as follows: physical neglect ≥8, physical abuse ≥8, emotional neglect ≥15, emotional abuse ≥10, sexual abuse ≥8 (Walker et al., 1999). Body Mass Index (BMI) was calculated after assessing self-reported height and body weight. BMI was dichotomized at 25 kg/m^2^ to classify overweight/obese individuals. Medication was obtained from clinical records.

### 2.3 Laboratory assessment

*Flow cytometry analysis:* Peripheral Blood Mononuclear cells (PBMCs) had been collected from the patients, frozen and stored in liquid nitrogen as described before (Counotte et al., 2018). Fluorescence-activated cell sorting (FACS) was performed on defrosted PBMCs, once washed with complete culture medium (RPMI-1640 culture medium plus 10% fetal calf serum (FCS) plus 1% penicillin/streptomycine). Recovery and viability of cells were determined by Trypan blue staining.

Staining A – Percentages of T cells (CD3^+^), T helper (CD3^+^CD4^+^) lymphocytes, T cytotoxic (CD3^+^CD8^+^) lymphocytes, natural killer cells (CD3^−^CD56^+^), and B cells (CD19^+^) were assessed by staining 50.000 PBMCs in a 8-color membrane staining (CD45, CD3, CD4, CD8, CD19, CD14, CD56 and CD15). Cell determinations from staining A are presented as frequency of PBMCs.

Staining B – Percentages of T helper cell subsets (Th1, Th2, Th17, T regulator cells) were determined by performing a 8-color (membrane and intracellular) staining on 1 x 10^6^ of PBMCs after a 4 h stimulation at 37 °C in RPMI-1640 culture medium with 50 ng/ml phorbol 12-myristrate 13-acetate (PMA; Sigma Aldrich, St. Louis, MO, USA) and 1.0 μg/ml ionomycin (Sigma) in the presence of Golgistop (BD Biosciences). T helper cell subsets were identified by their secreting cytokines: Th1 (CD3^+^CD4+IFNγ+), Th2 (CD3^+^CD4+IL4+), Th17 (CD3^+^CD4+IL17A+). T regulatory (Treg) cells were identified by their transcription factor FOXP3 (CD3^+^CD4^+^CD25hiFOXP3+). We also measured proportions of memory CD4^+^ T cells (CD3^+^CD4^+^CD45RO+) and naïve CD4^+^ T cells (CD3^+^CD4^+^CD45RO-); the latter was indicated by subtracting the proportion of memory CD4 T cells from the total T helper lymphocytes. T cell subsets of staining B were expressed as percentages of total lymphocytes, which could be reliably detected as a clear population in scatter properties after the 4-h culture. Also, when expressed as a percentage of total lymphocytes as found in staining A (the non-cultured sample), the outcomes did not alter.

Staining C – For more profound analysis of T helper and cytotoxic naïve/memory cell subsets a second vial of PBMCs was defrosted. Average recovery of cells after thawing was 73% and viability 93%. 1,5 x 10^6^ of PBMCs were stained with a cocktail of CD45-V500, CD45RA-BB515, CD3-Alexa Fluor700, CD4-PE-Cy7, CD8-BV786, CD197-BV421 (BD Biosciences), CD28-BV711, CD27-APC and CD57-PE (BioLegend) for 15 min at Room temperature, washed twice with PBS, pH 7.8 and subsequently stained with viability dye eFluor780 (Thermo Fisher Scientific). 500.000 events in a live/CD45 stopping gate were collected on a BD LSR Fortessa-4 laser instrument. Analysis was performed using FlowJo software. Quadrant gating on CD45RA and CD197 (CCR7) was used to define subsets of the CD4^+^ and CD8^+^ populations: naïve like (CD45RA + CD197+), central memory (CD45RA + CD197+), effector memory (CD45RA + CD197+) and effector memory RA (TEMRA; CD45RA + CD197-). The total CD8 and CD4 Tmemory populations were calculated by adding the respective T memory subpopulations Tcm, Tem, and TEMRA. The expression of CD27 and CD28 was assessed within each indicated T cell subset (Gating strategy is given in Supplementary Fig. 1). Subset determinations from staining C are presented as frequency of CD3^+^ T cells.

*CMV antibody titer determination:* Antibodies to CMV were measured using the Cytomegalovirus IgG ELISA kit manufactured by Demeditec Diagnostics GmbH, Germany, according to the instructions for use. Briefly, samples were diluted 1:101 and subsequently incubated together with four calibrators in a CMV antigen (strain AD-169) coated ELISA plate. Unbound material was removed during a washing step whereafter polyclonal rabbit anti-human IgG antibodies labeled with the enzyme HRP were added for incubation. After a final washing step, chromogen was added, and the resulting color development was measured spectrophotometrically. The values of the samples were calculated by interpolation from the calibrator curve ranging from 1 to 90 U/ml, with cut-off value of 10 U/ml. Values exceeding the highest calibrator were reported as such. Intra-assay precision reported by the manufacturer is within 9.6–12.2%, while clinical specificity and clinical sensitivity of the assay is 98% and 100%, respectively. CMV titers were dichotomized into qualitative data at 10 U/mL into CMV seropositive and CMV seronegative cases.

### 2.4 Statistics

For descriptive statistics, number and frequencies or median and interquartile range are reported. Chi-square test or Fisher's exact test were performed for contingency tables. For multiple group comparisons on continuous outcomes Kruskal-Wallis test was used, followed by pairwise Wilcox post-hoc test with Benjamini-Hochberg correction for multiple testing for single group comparisons. Non-parametric tests were chosen with regards to deviations from normal distribution and thus to demonstrate findings with a more conservative test. Adjusted multivariate analyses were performed using multiple linear regression to predict lymphocyte subset frequencies by diagnosis and CMV serostatus. Applying the central limit theorem, this approach was chosen to control for the potential confounders CA, age, sex, BMI. Further, the model was run twice investigating only additive main effects of diagnosis and CMV and the interaction effect of both variables. Due to the small percentage of missing values, mean imputation was used to replace missing values of cell frequencies, separately for MDD patients and HCs. All calculations and figures were done using R studio version 4.0.3.

## 3 Results

### 3.1 Demographics

Table 1 shows the population demographics of the investigated MDD population in staining A and B. Following inspection of the descriptive data, selected comparisons were tested for statistical significance: Women are overrepresented in both the HC and MDD CMV positive groups (χ^2^ = 13.82; p < 0.001), while the CMV titers are comparable between HC CMV positive cases and MDD CMV positive cases (W = 920; p = 0.35). Interestingly, MDD CMV positive cases had a lower BMI as compared to the MDD CMV negative cases (W = 1466; p = 0.02); they were also less overweight/obese (χ^2^ = 5.24; p = 0.02). Regarding childhood trauma, MDD CMV positive and MDD CMV negative cases had a similar prevalence (χ^2^ = 0.66; p = 0.42), however the MDD CMV positive cases were more severely traumatized (W = 2023.5; p = 0.04). Demographics of the staining C subsample can be found in Supplementary Table 1. Although numbers of patients and controls were less, similar patient and control characteristics were observed.

**Table 1:** Demographic and clinical characteristics of patients and healthy controls per CMV status (population staining A and B).

	HC CMV-	HC CMV+	MDD CMV-	MDD CMV+	Test statistic	p-value
n (%)	73 (64.6)	40 (35.4)	75 (59.1)	52 (40.9)	χ^2^ = 0.56	0.45
female, n (%)	46 (63.0)	32 (80.0)^a^	37 (49.3)	42 (80.8)^a^	χ^2^ = 17.94	<0.001
age (years), Md (IQR)	37 (20.88)	40.32 (26.55)	37 (19)	44.2 (15.675)	χ^2^ = 5.63	0.13
CMV titer (U/mL), Md (IQR)	4 (1.9)	58.9 (43.85)	3.7 (2.02)	59.59 (40.83)	χ^2^ = 170.08	<0.001
BMI, Md (IQR)	23.55 (4.97)	23.13 (4.39)	24.54 (5.03)	23.39 (4.34)^a^	χ^2^ = 9.62	0.02
overweight/obese, n (%)	21 (28.8)	13 (32.5)	35 (46.7)	13 (25.0)^a^	χ^2^ = 251.95	<0.001
CTQ sum score, Md (IQR)	31 (9)	30 (11.25)	33.5 (14.75)	42 (23)^a^	χ^2^ = 22.37	<0.001
childhood trauma present, n (%)	25 (34.2)	11 (27.5)	38 (50.7)	31 (59.6)	χ^2^ = 13.75	0.003
medicated (psychotrop.), n (%)	0 (0.0)	0 (0.0)	53 (94.6)	37 (92.5)		0.69

Notes. MDD Major depressive disorder; HC healthy controls; CMV cytomegalovirus; Md median; IQR interquartile range. Chi-square test or Fisher's exact test were performed for contingency tables, as well as Kruskal-Wallis tests for continuous outcomes.

a Indicating significant differences as described in the section 3.1 Demographics.

### 3.2 Lymphocyte subset frequencies in staining A in patients with and without CMV seropositivity

**Fig. 1:**
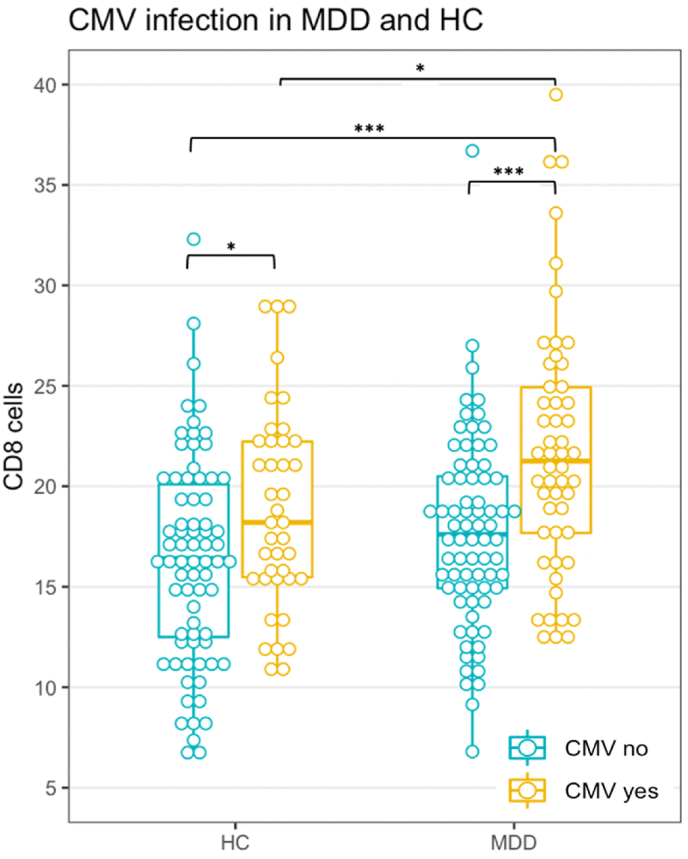
T cytotoxic cell frequency in MDD and HC. Notes. Staining A as frequency of PBMCs. See Table 2 for median and IQR. *p < 0.05; **p < 0.01; ***p < 0.001.

Table 2 shows the lymphocyte subset cell frequencies for HC and MDD patients with and without positive CMV antibodies. In staining A, differences between the groups were found for CD3^+^ T cells and CD8^+^ T cytotoxic cells. Here, in both HC and MDD this was due to an increase in CD8^+^ T cell frequency in CMV-seropositive individuals as compared to seronegative individuals. Fig. 1 displays the data of the CD8^+^ T cytotoxic cells. Although T cytotoxic cell frequencies were increased in both CMV-antibody positive HC and MDD patients, we found the increase in MDD patients significantly larger than in HC. Thus, main effects of diagnosis and CMV infection became apparent leading to the highest values in the MDD CMV-seropositive group as an additive effect. The adjusted multivariate analysis confirms the results regarding the diagnosis and CMV main effects with highly significant coefficients (R^2^ = 0.24; F(7,232) = 10.23; p < 0.001; see Supplementary Table 3). Adding the interaction term in a second model reduced the explained variance by the main effects (diagnosis: β = 1.18; p = 0.17, CMV: β = 3.12; p = 0.005) and was itself not significant (β = 1.76; p = 0.22), thus again supporting the presence of an additive effect.

**Table 2:** Staining A and B cell frequencies of lymphocyte subsets per diagnosis and CMV status.

	HC CMV-	HC CMV+	MDD CMV-	MDD CMV+	Test statistic	p-value
staining A						
CD3, Md (IQR)	57.1 (10.6)	63.7 (7.83)*	61 (11.5)	63.9 (11.2)*	χ^2^ = 16.89	<0.001
CD4, Md (IQR)	37.8 (9.2)	40.1 (8.85)	39.1 (9.55)	38.51 (11)	χ^2^ = 4.27	0.23
CD8, Md (IQR)	16.4 (7.6)	18.2 (6.75)*	17.6 (5.7)	21.25 (7.25)*	χ^2^ = 25.54	<0.001
NK, Md (IQR)	9 (4.97)	7.7 (5.21)	8.2 (5.22)	7.81 (5.65)	χ^2^ = 4.06	0.26
B, Md (IQR)	7.07 (3.14)	8.03 (3.03)	7.28 (2.76)	8.02 (3.97)	χ^2^ = 4.33	0.23
staining B						
Th naïve, Md (IQR)	28.9 (9.1)	28.55 (13.08)	25.8 (11)	22.3 (12.03)*	χ^2^ = 20.24	<0.001
Th memory, Md (IQR)	21.9 (8.1)	22.45 (8.18)	24.7 (9.05)*	24.61 (10.35)*	χ^2^ = 10.69	0.01
Th1, Md (IQR)	4.37 (2.21)	5.9 (3.59)*	4.36 (2.88)	5.42 (2.94)*	χ^2^ = 23.31	<0.001
Th2, Md (IQR)	0.47 (0.23)	0.43 (0.22)	0.41 (0.31)	0.44 (0.26)	χ^2^ = 6.90	0.08
Th17, Md (IQR)	0.30 (0.16)	0.26 (0.15)	0.32 (0.24)	0.29 (0.16)	χ^2^ = 3.97	0.26
Treg, Md (IQR)	1.87 (0.95)	1.84 (0.92)	2.12 (0.10)*	1.85 (0.69)	χ^2^ = 9.17	0.03

Notes. MDD Major depressive disorder; HC healthy controls; CMV cytomegalovirus; Md median; IQR interquartile range. Kruskal-Wallis tests were performed. *CD3 cell frequencies in the MDD and HC groups with CMV were significantly higher compared to HC without CMV (p = 0.002 in both cases). CD8 cell frequencies in the HC and MDD groups with CMV were significantly higher than their counterparts without CMV (p = 0.04 and p < 0.001, respectively), while the latter group also had increased cell frequencies compared to both HC groups (p < 0.001 and p = 0.04). Th naïve cell frequencies of the MDD group with CMV were significantly lower than all other groups (p < 0.001, p = 0.002, and p = 0.005, respectively). Th memory cell frequencies of the MDD groups with and without CMV were significantly higher than HC without CMV (p = 0.02 in both cases). Th1 cell frequencies were significantly increased in the CMV positive MDD and HC groups compared to their CMV negative counterparts (p = 0.004 in both cases). Treg cell frequencies of the MDD group without CMV were significantly higher than MDD patients with CMV (p = 0.03).

Table 2 also shows the data on NK and B cells. In the previous analysis of Schiweck et al. (2020) significantly higher B cells and lower NK cells in the entire group of MDD patients had been found, while our analysis shows that these differences cannot be explained by CMV-seropositivity.

### 3.3 Lymphocyte subset frequencies in staining B in CMV seropositive and seronegative patients

With regard to the subdivision of the CD4^+^ T helper populations (staining B) we found significantly higher Th1 cell frequencies in both the CMV-seropositive HC and MDD patients (Table 1). In the previous analysis of Schiweck et al. (2020) differences in Th1, Th2, and Th17 were not found between MDD patients and HC, while there was a trend for a difference in T regulatory cells. Taking the CMV-antibody state of patients and controls into account, the present analysis shows that particularly seronegative MDD patients show higher frequencies of T regulatory cells (Table 1).

**Fig. 2:**
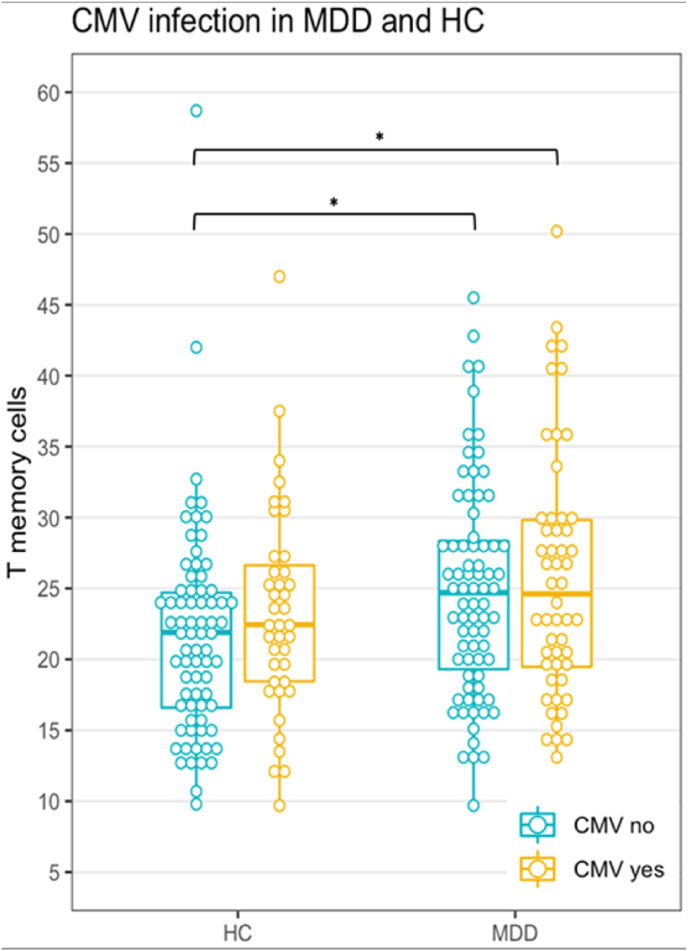
T helper memory cell frequency in MDD and HC. Notes. Staining B as frequency of total lymphocytes. See Table 2 for median and IQR. *p < 0.05.

**Fig. 3:**
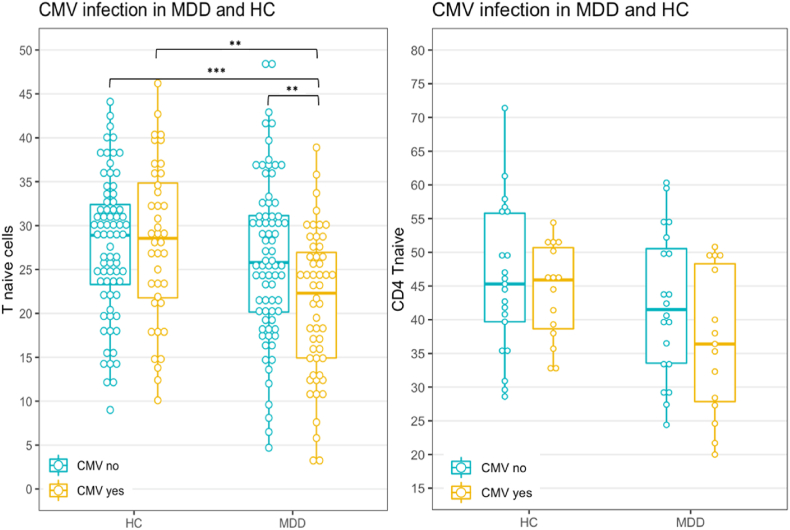
T helper naïve cell frequency in MDD and HC. Notes. Staining B as frequency of total lymphocytes (left), staining C as frequency of CD3^+^ (right). See Table 2 and Table 4 for median and IQR. **p < 0.01; ***p < 0.001.

In staining B we also found that the frequency of CD4^+^ T helper memory cells (CD45RO + cells) was raised in MDD patients irrespective of the CMV-antibody state (Table 1, Fig. 2). With regard to the CD4^+^ T helper naïve cells (CD45RO-) a significantly reduced frequency was only found in the CMV-seropositive MDD patients pointing towards an important role of CMV infection (Table 1, Fig. 3). Based on these findings and also based on the previous conclusions of Schiweck et al. (2020), we analyzed the memory and naïve populations of the CD8^+^ and CD4^+^ T cell populations in more detail using another staining strategy, i.e., staining C.

### 3.4 CD4 and CD8 lymphocyte subpopulation frequencies in CMV seropositive and seronegative patients and HC

**Fig. 4:**
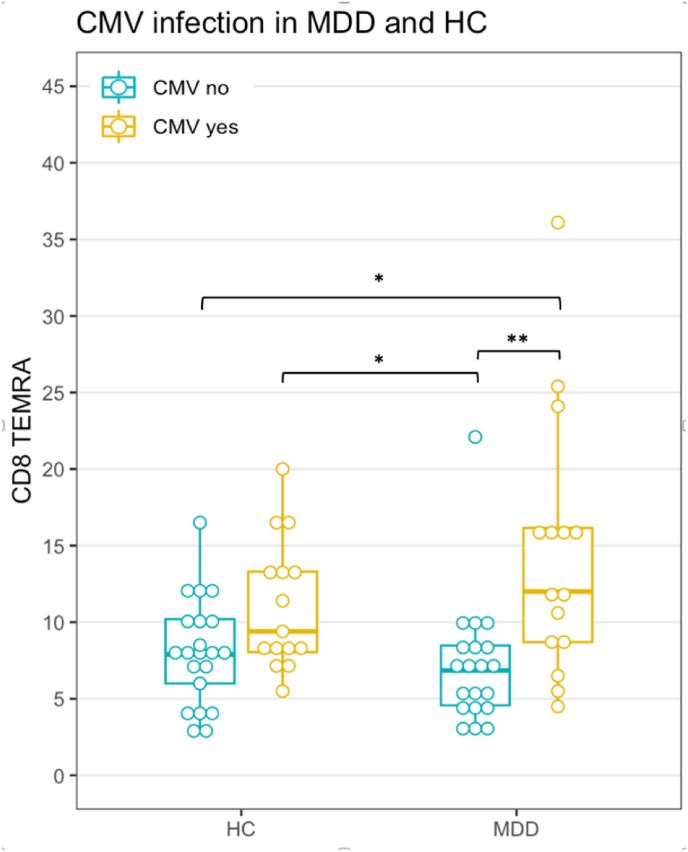
T cytotoxic cell TEMRA subpopulation frequency in MDD and HC. Notes. Staining C as frequency CD3^+^. See Table 3 for median and IQR. *p < 0.05; **p < 0.01; ***p < 0.001.

*CD8*^*+*^*T cells in CMV seropositive and seronegative MDD patients and HC:* Staining C data shows that the exaggerated increase of CD8^+^ cytotoxic T cells in CMV-antibody positive MDD patients (as illustrated in Fig. 1) was mainly due to a statistically significant increase in memory cells (although naïve cells were also increased, be it not significantly; Table 3). This increase in memory cells was driven by the CD8 TEMRA subpopulation (Table 3 and Fig. 4). Late stage CD27^−^CD28^−^ TEMRA cells were similarly increased in CMV seropositive HC and MDD patients (Supplementary Table 2).

**Table 3:** T cytotoxic cell subpopulations in MDD and HC as frequency of CD3+cells (staining C).

	HC CMV-	HC CMV+	MDD CMV-	MDD CMV+	Test statistic	p-value
CD8 T naive, Md (IQR)	14.7 (8.8)	12.5 (6.8)	15.6 (6.3)	19 (9.3)	χ^2^ = 2.36	0.50
CD8 T memory, Md (IQR)	11.4 (6.2)	14.2 (6.5)	9.62 (6.8)	18.2 (11.4)*	χ^2^ = 10.6	0.01
CD8 Tcm, Md (IQR)	0.62 (0.47)	0.77 (0.72)	0.65 (0.67)	0.45 (0.75)	χ^2^ = 2.24	0.52
CD8 Tem, Md (IQR)	2.43 (2.35)	2.22 (1.72)	1.95 (2.34)	1.8 (2.61)	χ^2^ = 0.45	0.93
CD8 TEMRA, Md (IQR)	7.9 (4.2)	9.4 (5.25)	6.85 (3.9)	12 (7.45)*	χ^2^ = 15.66	0.001

Notes. As frequency of CD3^+^. *CD8 T memory cell frequencies of the MDD group with CMV were increased compared to their counterparts without CMV as a strong statistical trend (p = 0.05) and CD8 TEMRA cell frequencies of the MDD group with CMV were significantly higher than their counterparts without CMV (p = 0.01).

*CD4*^*+*^*T helper memory cell frequencies in CMV seropositive and seronegative MDD patients and HC:* In staining B T helper memory cell frequencies were increased in MDD patients as compared to HC, irrespective of the CMV-antibody state (Fig. 2). In fact, differences between frequencies of CD4^+^ T helper memory cells were negligible between seropositive and seronegative subjects. Multivariate adjusted analysis confirms these results (R^2^ = 0.30; F(7,232) = 13.95; p < 0.001) with a highly significant main effect for diagnosis (Supplementary Table 4). Adding the interaction term of diagnosis and CMV was not significant (ß = −1.50; p = 0.43) and did not alter the explained variance to a relevant degree. From staining C data it becomes apparent that the increase in CD4^+^ T helper memory cells in MDD patients was due to a slightly (and not significant) different cell apportioning in CMV seronegative and seropositive patients (Table 4): In seronegative MDD patients T central memory cell frequency was the highest, while in seropositive MDD patients T effector memory cell frequency was the highest. The TEMRA cell frequency was significantly increased in both seropositive HC and MDD patients as compared to their seronegative counterparts. The frequencies of terminally differentiated CD27^−^CD28^−^ TEMRA cells were also equally raised in both seropositive HC and MDD patients as compared to their seronegative counterparts (Supplementary Table 2).

**Table 4:** Staining C cell frequencies of the CD4 T naïve and memory cell subsets.

	HC CMV-	HC CMV+	MDD CMV-	MDD CMV+	Test statistic	p-value
CD4 Tnaive, Md (IQR)	45.3 (16.1)	45.9 (12.05)	41.5 (17)	36.4 (20.45)	χ^2^ = 6.00	0.11
CD4 Tmemory, Md (IQR)	21.35 (9.7)	21.5 (3.5)	24.7 (10.2)	21.3 (10.8)	χ^2^ = 2.61	0.46
CD4 Tcm, Md (IQR)	12.2 (6.8)	12.9 (4.8)	14.9 (6.95)	10.8 (6.2)	χ^2^ = 4.75	0.19
CD4 Tem, Md (IQR)	6.05 (3.26)	5.54 (3.86)	5.85 (6.91)	8.23 (4.22)	χ^2^ = 2.71	0.44
CD4 TEMRA, Md (IQR)	1.23 (1.35)	2.4 (1.56)*	1.56 (0.78)	2.44 (2.11)*	χ^2^ = 15.23	0.002

Notes. As frequency of CD3^+^. *CD4 TEMRA cell frequencies of the HC and MDD groups with CMV were significantly higher than their counterparts without CMV (p = 0.04 and p = 0.006, respectively).

*CD4*^*+*^*T helper naïve cell frequencies in CMV seropositive and seronegative MDD patients and HC:* In staining B the CD4^+^ T helper naïve (CD45RO-) cell frequency was decreased in CMV seropositive MDD patients as compared to the three other groups (Fig. 3 left and Table 2), while in seropositive HC CD4^+^ T helper naïve (CD45RO-) cell frequency was not decreased. Thus, reduced percentages of T helper naïve cells are an interactive effect of diagnosis and CMV seropositivity. The same pattern emerged from staining C (Fig. 3 right); however, differences did not reach statistical significance, most likely due to lacking statistical power with the low number of samples we were able to test in this analysis. The adjusted multivariate analysis confirms these results (R^2^ = 0.18; F(8,231) = 6.32; p < 0.001) after adding the interaction term by showing the significant diagnosis by CMV interaction effect on T helper naïve cell frequency (Supplementary Table 5). In the additive model, a significant diagnosis main effect and a trend for CMV main effect emerged (diagnosis: ß = −3.32; p = 0.004, CMV: ß = −2.47; p = 0.11).

### 3.5 CMV prevalence in relation to childhood adversity

**Fig. 5:**
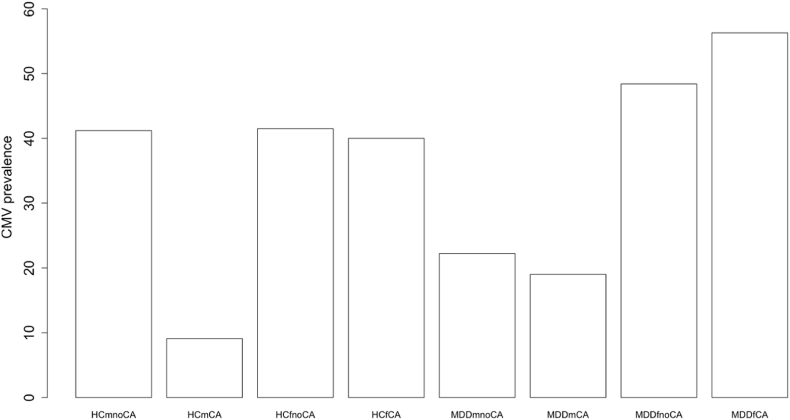
Prevalence of CMV positivity per diagnosis, sex, and presence of CA. Notes. Frequency of CMV positive cases in each subgroup. *CMV* cytomegalovirus; *HC* healthy control; *MDD* major depressive disorder; *m* male; *f* female; *noCA* no childhood adversity; *CA* childhood adversity. Exact percentages are (from left to right): 41.2; 9.1; 41.5; 40; 22.2; 19; 48.4; 56.3.

The highest prevalence of CA is found in CMV-antibody positive MDD patients (Table 1). Based on a previous publication by Ford et al. (2020) in which a lower prevalence was found in males, we also took sex into account. Fig. 5 shows the prevalence of CMV seropositivity in healthy controls and MDD patients per sex and with and without CA. Females had higher rates of seropositivity as compared to males in MDD patients and as a trend HC (MDD: χ^2^ = 11.61; p < 0.0001, HC: χ^2^ = 2.74; p = 0.10). An overall significant difference between HC and MDD patients was not found (χ^2^ = 0.56; p = 0.45). With regard to CA, a higher prevalence of CMV seropositivity was found numerically but not statistically significant in MDD patients with CA in both females and males compared to their HC counterparts with CA (females: χ^2^ = 1.15; p = 0.28, males: χ^2^ = 0.05; p = 0.82). Female MDD patients with a history of CA showed the highest percentage of CMV seropositivity, i.e., 56.3%.

### 3.6 Childhood adversity and T cell apportioning

Supplementary Table 6 gives the results of cell frequencies of the analyzed lymphocyte populations for cases with and without CA. In fact, relevant differences in the tested lymphocyte subpopulations could not be found. Interestingly, in staining B CD4^+^ T helper memory cell (CD45RO+) frequency was clearly and significantly increased in MDD patients without CA as compared to HC without CA. This supports the idea that the increased memory cell frequency may be an effect of the diagnosis and not of the CA status. CD4^+^ T helper naïve cell (CD45RO-) frequency was not altered with respect to CA.

## 4 Discussion

We previously described higher percentages of circulating T helper memory cells in the EU-MOODINFLAME cohort of MDD patients and we interpreted the findings as pointing towards a premature aging of the T cell compartment in MDD patients (Schiweck et al., 2020). Here we studied such putative accelerated aging in more detail, focusing on the role of CMV-antibody seropositivity (as a sign of chronic CMV infection) and involving better defined naïve and memory T cell subpopulations, including late stage differentiated memory T cells. Differences in parameters of T cell aging were investigated with respect to disease state and CMV seropositivity. Our main findings are: 1) A higher inflation of memory CD4^+^ T helper cells in CMV seronegative MDD patients as compared to seronegative healthy controls. 2) Reduced numbers of naïve CD4^+^ T helper cells in CMV seropositive patients, while such reductions were not found in CMV seropositive HC. 3) A higher inflation of memory CD8^+^ T cytotoxic cells in CMV seropositive MDD cases as compared to CMV seropositive HC, particularly of the TEMRA cells.

We interpret these findings as showing that the previously found premature senescence of the T cell compartment as described by Schiweck et al. (2020) is a type of senescence driven by a „double hit” being the MDD disease state and the state of chronic CMV infection. The state of MDD is mainly associated with the high inflation of the memory CD4^+^ T helper compartment, while the state of MDD together with a chronic CMV infection (a double hit“) is associated with the high inflation of the memory CD8^+^ cytotoxic T cell compartment (as an additive effect) and the reduced percentage of naive CD4^+^ T cells (as an interaction effect). Several possibilities may explain these findings. Enhanced recurrent activations of the T cell system (perhaps due to an enhanced susceptibility to infections other than CMV) might have led to the increased frequencies of the CD4^+^ and CD8^+^ memory cells with a reciprocal loss of the naïve CD4^+^ T cells. The loss of the naive CD4^+^ T cells might also be due to a premature aging-related atrophy of the thymus with an insufficient output of the CD4^+^ T cells or to an increased apoptosis of these CD4^+^ cells after leaving the thymus. Thymic atrophy is a normal mechanism of the T cell senescence process (Ongrádi and Kövesdi, 2010; Miller, 2010), while an enhanced apoptosis of recent thymic emigrants has been described as a genetically determined defect in the autoimmune model of the BB rat (Sommandas et al., 2007). Our study also shows that CMV seropositivity (and not the state of MDD) was associated with another aspect of aging, namely a higher percentage of TEMRA and late stage CD27^−^CD28^−^ TEMRA cells in both HC and MDD patients. Therefore, this type of senescence characterized by exhausted T cells was entirely due to the state of chronic CMV infection in both HC and MDD patients.

Ford et al. (2020) also found a premature aging of the T cell compartment in MDD patients. However, they found it to be entirely explainable by CMV seropositivity, while refuting a role of the MDD state and a “double hit” effect in T cell aging (Ford et al., 2020). Differences in results between this study and ours may lie in the investigated study populations. Comparing their patient and control groups with the ones analyzed in our study some similarities (such as the in general young age) but also some remarkable differences can be detected. While similar overall prevalence of around 40–50% CMV seropositivity in HC and patients were present in both study populations, Ford et al. (2020) detected a very low prevalence of 16% seropositivity in MDD males and a very high prevalence of 71% in MDD females. Our sample did not show such an extreme sex difference in MDD, although MDD males had a lower prevalence of CMV seropositivity. However, this was in line with the well-accepted higher prevalence of seropositivity for females in general and present in our HC (see Fig. 5). Another difference between the two study populations is the BMI of patients and controls. While in the US population subjects had a BMI ranging from 26 kg/m^2^ to 29 kg/m^2^ (Ford et al., 2020), the European population had a BMI ranging from 24 to 25 kg/m^2^. Moreover, their CMV seropositive subjects had increased weight (Ford et al., 2020), while our seropositive individuals had reduced weight. Since obesity has well known effects on immune aging (Shirakawa and Sano, 2021), we used it as a covariate in the adjusted analyses. However, no significant effects for BMI were found. Thus, it is difficult to attribute differences in study results simply to the BMI.

This also indicates that other differences such as cultural, dietary, microbial, and geographical differences between the US and Europe must have played a role in the discrepancies. Last, in the cohort of Ford et al. (2019) early life stress was associated with a higher prevalence of CMV-seropositivity. We detected a trend for such association, particularly in male MDD patients with CA who showed more CMV seropositive cases than HC with CA. However, this observation should be interpreted with caution due to the low number of healthy males with childhood trauma in our cohort (only 1 out of 11 males with childhood trauma was positive for CMV). Anyhow, CMV seropositive patients had experienced worse early life stress in general, i.e., had higher CTQ sum scores. Regarding CA and T cell aging, we were unable to find any relevant associations. On the contrary, our analysis showed a clear and significant difference according to the MDD disease state in cases without CA for the CD4^+^ T helper memory and naïve cells.

Noteworthy, we cannot rule out effects of chronic infections other than CMV for the associations of the MDD disease state with the increase in frequency of the CD4^+^ and CD8^+^ T memory cells and the reduction in frequency of CD4^+^ T naïve cells presented in this study. It is possible that our MDD patients might have higher prevalence of other chronic infections influencing T cell aging, such as toxoplasma, EBV, herpes simplex virus and others, although a recent large scale Finnish study did not find such higher prevalence (Markkula et al., 2020). Future studies should take the presence of such chronic infections into account and/or find genetic polymorphisms related to the here found aspects of T cell aging. Also, the role of current chronic stress should not be neglected, since it is known that such stress plays a role in immune aging (Bauer et al., 2015).

## 5 Limitations

The present study has several methodological limitations. With the cross-sectional study design no causality can be established and it is not possible to measure whether the differences in cell frequencies between the study groups are long-lasting. Further, in this study convenience sampling was used which limits the external validity of study results as the study population may not be representative of the general MDD population. However, the inclusion of multiple international sites increases generalizability of study results. Another drawback is the small sample size in the staining C population. Consequently, negative results may be due to type II error, especially since no sample size calculation was performed for this analysis. This study presents secondary explorative analyses in the project where the sample size was determined by availability of material for additional determinations such as CMV-antibody status. Last, three types of potential confounders were not considered in this work: First, it was not possible to control for duration of the CMV-seropositive state and we based our analysis on the determination of IgG anti-CMV titers and did not test IgM anti-CMV titers. This would have informed on potential recent acute exacerbations of the CMV infection. Second, data was pooled from three sites where potential differences between sites may exist. Third, the vast majority of patients was medicated by a variety of drugs with high variability of multiple medication usage, which was not assessed systematically.

## 6 Conclusion

Despite these limitations we conclude that MDD patients show several signs of a CMV independent MDD state associated premature T cell aging, such as a CMV independent increase in CD4^+^ T memory cells and a latent naïve CD4 T-cell reduction and a latent CD8^+^ T-cell increase, these two latent T cell senescence abnormalities only becoming evident after CMV infection (double hit). Whether the here reported CMV independent and MDD state associated T cell senescence abnormalities are due to an intrinsic genetic predisposition or due to environmental influences other than a chronic CMV infection needs further exploration.

## Funding

This work was supported by the European Commission: EU 7th Framework program (grant number EU-FP7-CP-IP-2008-222963) and Horizon 2020 (grant number H2020-SC1-2016-2017/H2020-SC1-2017-Two-Stage-RTD) grants were received by HAD, Erasmus Medical Center Rotterdam. The funding source had no role in the study design, the data collection, the analysis and interpretation of data, the manuscript writing, and in the decision to submit the article for publication.

## Submission declaration

The present work has not been published previously, has not been submitted to another journal while under consideration for Brain, Behavior, and Immunity, and will not be published elsewhere upon acceptance. The manuscript in its current form has been approved by all co-authors.

## Declaration of competing interest

MSS, MI and AW declare no potential conflict of interest. GA received personal fees from Janssenoutside the submitted work. RB is an employee of Advanced Practical Diagnostics BVBA. RM declares personal fees from Otsuka/Lundbeck outside the submitted work. NM was supported by the foundation ‘Immunität und Seele’. HAD is the coordinator of the EU-MOODSTRATIFICATION project and declares no further potential conflict of interest.

## Data Availability

Data will be made available on request.
