# Supplementary material for: Premature T cell aging in major depression: A double hit by the state of disease and cytomegalovirus infection
Source: Brain Behav Immun Health. 2023 Feb 27;29:100608. doi: 10.1016/j.bbih.2023.100608 (PMC9995284; doi:10.1016/j.bbih.2023.100608)
Supplement: Multimedia component 1 [file mmc1.docx]

Supplementary material

Supplementary table 1. Demographic and clinical characteristics of a subset of patients and healthy controls per CMV status – staining C subsample.

|  | HC CMV- | HC CMV+ | MDD CMV- | MDD CMV+ | Test statistic | p-value |
| --- | --- | --- | --- | --- | --- | --- |
| n (%) | 21 (58.3) | 15 (41.7) | 20 (57.1) | 15 (42.9) | χ^2^<0.001 | 1 |
| female, n (%) | 13 (61.9) | 10 (66.7) | 13 (65.0) | 11 (73.3) | χ^2^=0.53 | 0.91 |
| age (years), Md (IQR) | 38.5 (16.2) | 31.08 (18.0) | 32.4 (16.71) | 35.8 (12.39) | χ^2^=0.14 | 0.99 |
| CMV titer (U/mL), Md (IQR) | 4 (1) | 41.4 (47.55) | 3.52 (1.39) | 63.8 (42.52) | χ^2^=51.74 | <0.001 |
| BMI, Md (IQR) | 23.55 (3.33) | 21.47 (5.01) | 25.22 (11.27) | 22.34 (4.86) | χ^2^=7.58 | 0.06 |
| overweight/obese, n (%) | 5 (23.8) | 4 (26.7) | 11 (55.0) | 3 (20.0) | χ^2^=79.95 | <0.001 |
| CTQ sum score, Md (IQR) | 31 (9) | 29 (6) | 34 (14) | 38 (16.5) | χ^2^=12.53 | 0.006 |
| childhood trauma present, n (%) | 7 (33.3) | 2 (13.3) | 9 (45.0) | 8 (35.3) | χ^2^=6.01 | 0.11 |
| medicated (psychotrop.), n (%) | 0 (0.0) | 0 (0.0) | 15 (88.2) | 13 (86.7) |  | 1.00 |

Notes. MDD Major depressive disorder; HC healthy controls; CMV cytomegalovirus; Md median; IQR interquartile range. Chi-square test or Fisher’s exact test were performed for contingency tables, as well as Kruskal-Wallis tests for continuous outcomes.

Supplementary table 2. Late stage differentiated cell frequencies (staining C).

|  | HC CMV- | HC CMV+ | MDD CMV- | MDD CMV+ | Test statistic | p-value |
| --- | --- | --- | --- | --- | --- | --- |
| CD4 Tem 27-28-,  MD (IQR) | 0.007 (0.007) | 0.27 (0.58)* | 0.007 (0.02) | 0.67 (0.73)* | χ^2^=38.66 | <0.001 |
| CD4 TEMRA 27-28-, Md (IQR) | 0.009 (0.01) | 0.76 (1.73)* | 0.02 (0.02) | 0.80 (1.60)* | χ^2^=45.17 | <0.001 |
| CD8 Tem 27-28-,  MD (IQR) | 0.08 (0.16) | 0.25 (0.66) | 0.07 (0.12) | 0.21 (0.67) | χ^2^=8.69 | 0.03 |
| CD8 TEMRA 27-28-, Md (IQR) | 1.05 (1.54) | 4.21 (5.46)* | 0.88 (1.08) | 5.25 (7.26)* | χ^2^=35.14 | <0.001 |

Notes. MDD Major depressive disorder; HC healthy controls; CMV cytomegalovirus; Md median; IQR interquartile range. *For CD4 Tem 27-28- cells, CD4 TEMRA 27-28- cells, and CD8 TEMRA 27-28- cells counts of the HC and MDD groups with CMV were significantly higher compared to their counterparts without CMV (p<0.001 in both cases).

Supplementary table 3. Adjusted analysis of additive effects for CD8+ cell counts.

| predictor | ß-coefficient | SE | t | p |
| --- | --- | --- | --- | --- |
| diagnosis | 1.83 | 0.68 | 2.68 | 0.008 |
| CMV status | 3.85 | 0.92 | 4.20 | <0.001 |
| CA status | 1.04 | 0.85 | 1.23 | 0.22 |
| age | -0.14 | 0.03 | -5.05 | <0.001 |
| sex | -0.93 | 0.73 | -1.23 | 0.20 |
| BMI | -0.12 | 0.08 | -1.56 | 0.12 |
| CMV*CA | 0.37 | 1.36 | 0.28 | 0.78 |

Notes. *SE* standard error; *CMV* cytomegalovirus; *CA* childhood adversity.

Supplementary table 4. Adjusted analysis of additive effects for Th memory cell counts.

| predictor | ß-coefficient | SE | t | p |
| --- | --- | --- | --- | --- |
| diagnosis | 3.06 | 0.91 | 3.38 | <0.001 |
| CMV status | -0.62 | 1.22 | -0.51 | 0.61 |
| CA status | -2.43 | 1.13 | -2.16 | 0.03 |
| age | 0.32 | 0.04 | 8.80 | <0.001 |
| sex | 2.06 | 0.97 | 2.13 | 0.03 |
| BMI | -0.05 | 0.10 | -0.52 | 0.61 |
| CMV*CA | 0.68 | 1.80 | 0.38 | 0.71 |

Notes. *SE* standard error; *CMV* cytomegalovirus; *CA* childhood adversity.

Supplementary table 5. Adjusted analysis of interactive effect for Th naïve cell counts.

| predictor | ß-coefficient | SE | t | p |
| --- | --- | --- | --- | --- |
| diagnosis | -1.05 | 1.42 | -0.74 | 0.46 |
| CMV status | 0.06 | 1.79 | 0.03 | 0.97 |
| CA status | 0.52 | 1.40 | 0.38 | 0.71 |
| age | -0.16 | 0.05 | 3.62 | <0.001 |
| sex | 3.24 | 1.20 | 2.71 | 0.007 |
| BMI | 0.04 | 0.13 | 0.29 | 0.78 |
| diagnosis*CMV | -6.09 | 2.33 | -2.62 | 0.009 |
| CMV*CA | 0.70 | 2.30 | 0.30 | 0.76 |

Notes. *SE* standard error; *CMV* cytomegalovirus; *CA* childhood adversity.

Supplementary table 6. Staining A, B, and C cell counts of lymphocyte subsets per diagnosis and CA status.

|  | HC CA- | HC CA+ | MDD CA- | MDD CA+ | Test statistic | p-value |
| --- | --- | --- | --- | --- | --- | --- |
| staining A |  |  |  |  |  |  |
| CD3, Md (IQR) | 59.6 (9.1) | 59.6 (12.25) | 60.25 (11.98) | 63.4 (11.8) | χ^2^=6.38 | 0.09 |
| CD4, Md (IQR) | 39.2 (8.8) | 37.75 (12.25) | 37.65 (10.68) | 39.3 (9.3) | χ^2^=2.07 | 0.56 |
| CD8, Md (IQR) | 17.1 (6.8) | 16.45 (8.2) | 17.95 (6.58) | 19.2 (7.5)* | χ^2^=8.13 | 0.04 |
| NK, Md (IQR) | 8.21 (5.05) | 9.41 (5.28) | 8.24 (5.37) | 7.79 (4.34) | χ^2^=5.67 | 0.13 |
| B, Md (IQR) | 7.08 (3.29) | 7.67 (2.8) | 7.14 (3.11) | 7.85 (3.4) | χ^2^=3.03 | 0.39 |
| staining B |  |  |  |  |  |  |
| Th naïve, Md (IQR) | 29.1 (8.6) | 24.8 (15.83) | 21.6 (11.79)* | 26 (11.8) | χ^2^=14.37 | 0.002 |
| Th memory, Md (IQR) | 22.5 (8.7) | 20.35 (7.25) | 25.2 (11.05)* | 23.3 (9.9) | χ^2^=11.12 | 0.01 |
| Th1, Md (IQR) | 4.89 (3.14) | 4.53 (2.94) | 5.52 (3.38) | 4.75 (2.79) | χ^2^=5.60 | 0.13 |
| Th2, Md (IQR) | 0.48 (0.25) | 0.42 (0.19) | 0.41 (0.19) | 0.44 (0.31) | χ^2^=4.48 | 0.21 |
| Th17, Md (IQR) | 0.30 (0.16) | 0.23 (0.14) | 0.32 (0.22) | 0.27 (0.18) | χ^2^=3.77 | 0.29 |
| Treg, Md (IQR) | 1.89 (0.89) | 1.72 (0.7) | 2.00 (0.69) | 2.05 (1.01) | χ^2^=3.62 | 0.31 |
| staining C |  |  |  |  |  |  |
| CD4 Tnaive, Md (IQR) | 45.9 (11.2) | 45.3 (20.6) | 37.25 (18.8) | 40.6 (16.2) | χ^2^= 4.40 | 0.22 |
| CD4 Tmemory, Md (IQR) | 21.53 (5.32) | 20.04 (11.86) | 24.74 (11.11) | 22.77 (9.78) | χ^2^= 2.53 | 0.47 |
| CD4 Tcm, Md (IQR) | 12.9 (5.4) | 11.8 (5.7) | 13.25 (11.83) | 14 (7.6) | χ^2^= 0.11 | 0.99 |
| CD4 Tem, Md (IQR) | 5.54 (3.26) | 6.05 (4.82) | 6.21 (6.21) | 8.2 (4.91) | χ^2^= 2.12 | 0.55 |
| CD4 TEMRA, Md (IQR) | 1.72 (1.53) | 1.31 (1.93) | 1.63 (1.17) | 1.94 (0.85) | χ^2^= 1.32 | 0.73 |
| CD8 Tnaive, Md (IQR) | 14.1 (6.9) | 17.8 (10.5) | 15.25 (7.3) | 18.9 (7.2) | χ^2^= 4.93 | 0.18 |
| CD8 Tmemory, Md (IQR) | 13.41 (6.36) | 9.92 (3.64) | 13.76 (8.38) | 9.81 (10.32) | χ^2^= 2.68 | 0.44 |
| CD8 Tcm, Md (IQR) | 0.64 (0.48) | 0.62 (0.27) | 0.73 (0.99) | 0.61 (0.53) | χ^2^= 0.70 | 0.87 |
| CD8 Tem, Md (IQR) | 2.62 (2.11) | 1.57 (1.15) | 2.49 (2.91) | 1.79 (0.97) | χ^2^= 4.75 | 0.19 |
| CD8 TEMRA, Md (IQR) | 8.3 (5.15) | 7.9 (3.1) | 8.2 (5.8) | 8.5 (6.5) | χ^2^= 1.03 | 0.79 |

Notes. Staining A as frequency of PBMCs; staining B as frequency of total lymphocytes; staining C as frequency of CD3+. *T helper memory cells counts of the MDD group without CA were significantly higher compared to HC without CA (p=0.04); T helper naïve cell counts of the MDD group with CA were significantly lower than HC without CA (p=0.001); CD8 cell counts of the MDD group with CA were higher compared to HC without CA as a statistical trend (p=0.07).


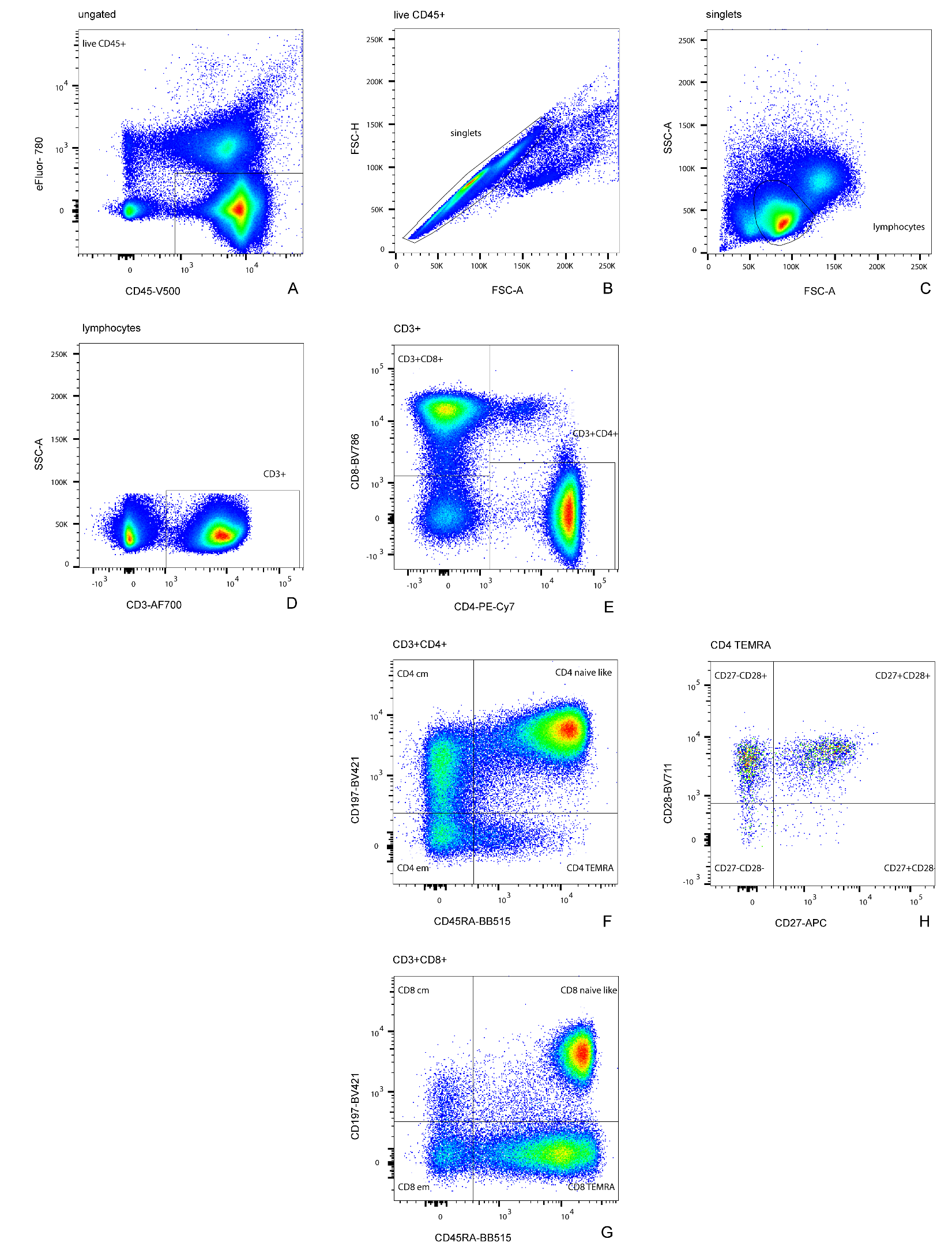
Supplementary Figure 1. Gating strategy.

Notes. A. Selection of live (CD45+) leukocytes. Gating was performed on CD45+/eFluor780 negative events, excluding dead cells, platelets, erythrocytes and cell debris. B. Selection of single cells within the live CD45+ gate, based on forward scatter area and height properties.

Notes (continued). C. Selection of lymphocytes within the singlets gate, based on forward scatter and side scatter areas. D. Selection of total T cells within the lymphocytes gate. Cells positive for CD3 were selected. E. Selection of T-helper and T-cytotoxic cells within the T cell CD3+ gate. CD3+CD4+ and CD3+CD8+ cells were selected. F. Definition of naïve like, central memory, effector memory and TEMRA subsets, within the T-helper cells, based on differental expression of CD45RA and CD197. G. Definition of naïve like, central memory, effector memory and TEMRA subsets, within the T-cytotoxic cells, based on differental expression of CD45RA and CD197 (CCR7). H. Quadrant gating of CD4 TEMRA cells reveals CD27+CD28+, CD27-CD28+, CD27-CD28- and CD27+CD28- sub-popula.
